# Potential of Circulating miRNA Biomarkers and Exosomes for Early Pregnancy Diagnoses in Cattle

**DOI:** 10.3390/ani14111592

**Published:** 2024-05-28

**Authors:** Chiaki Ninomiya, Hitomi Yoshino, Toshina Ishiguro-Oonuma, Kosuke Iga, Tomomi Kanazawa, Toru Takahashi, Keiichiro Kizaki

**Affiliations:** 1 Cooperative Department of Veterinary Medicine, Faculty of Agriculture, Iwate University, 3-18-8 Ueda, Morioka 020-8550, Iwate, Japanoonumat@iwate-u.ac.jp (T.I.-O.); tomomik@iwate-u.ac.jp (T.K.); tatoru@iwate-u.ac.jp (T.T.); 2 Graduate School of Veterinary Sciences, Iwate University, 3-18-8 Ueda, Morioka 020-8550, Iwate, Japan; a3124006@iwate-u.ac.jp; 3 Institute of Livestock and Grassland Science, National Agriculture and Food Research Organization, Ikenodai 2, Tsukuba 305-8602, Ibaraki, Japan; kosukei@affrc.go.jp

**Keywords:** biomarker, cattle, extracellular vesicle, microRNA, pregnancy

## Abstract

**Simple Summary:**

The early diagnosis of pregnancy in cattle is important for improving farm productivity, and a simple and early diagnostic method is needed. This study aimed to identify circulating microRNAs (miRNAs) that could serve as biomarkers for the early diagnosis of pregnancy in cattle. When the plasma levels of seven circulating miRNAs were examined, the values for six of the seven miRNAs were not significantly different between the pregnant and non-pregnant groups; however, miR-126-3p levels were significantly lower in the pregnant group. For the six miRNAs that did not differ significantly, the trend was toward higher levels during pregnancy than during non-pregnancy in the same individuals. These results suggest that circulating miR-126-3p can be used as a biomarker for early pregnancy diagnosis in cows and that some miRNAs could be used as indicators for pregnancy/non-pregnancy determination by accounting for individual differences.

**Abstract:**

Circulating microRNAs (miRNAs) were investigated as biomarkers for the diagnosis of early pregnancy in cattle. The levels of prospective miRNA biomarkers and the features of extracellular vesicles (EVs) in the blood were evaluated. In Study 1, plasma samples from cows 21 days after artificial insemination (AI) were examined using RT-qPCR to determine the levels of seven circulating miRNAs. Only the levels of miR-126-3p were significantly lower in the pregnant group than in the non-pregnant group. In Study 2, among individuals not pregnant at the first AI, the miRNA levels were compared between the individuals pregnant at the second AI and those who remained non-pregnant. The miR-25 levels were significantly higher in the pregnant group at the second AI than in the pregnant group at the first AI; miR-19b, miR-27b, and miR-29a levels were also high. In the non-pregnant group, changes were absent in the miRNA levels in the same individual between the first and second AIs. In Study 3, Western blotting and RT-qPCR showed the presence of miRNAs in EVs and their levels were lower than in plasma. Thus, circulating miR-126-3p may serve as a biomarker for the diagnosis of early pregnancy in cattle. In addition, the expression of some miRNAs tended to be higher during pregnancy than during non-pregnancy in the same individual, suggesting their potential as an index to determine pregnancy and non-pregnancy rates using a comparative method.

## 1. Introduction

Shortening the days open is of significant importance to animal productivity. The early detection of pregnancy is crucial in cattle because it allows for efficient subsequent insemination within 21 days of artificial insemination (AI) [1]. Ultrasonography, rectal palpation, blood progesterone measurement, and estrus detection are the primary methods used for pregnancy diagnosis in cattle [2,3,4]. Rectal palpation can be performed over 30 days post-AI, whereas ultrasonography can be performed between 25 and 27 days post-AI but requires skilled professionals for accurate diagnosis [5]. The progesterone test is problematic because it is positive even in non-pregnant cows with a prolonged luteal phase [2,3,4]. However, an easily accessible and non-invasive method for early pregnancy diagnosis is required. Recently, new methods for pregnancy diagnosis have been suggested, including the gene expression analysis of peripheral leukocytes, which allows for a diagnosis within 21 days of pregnancy [6,7,8,9,10,11,12,13]. On the other hand, the measurement of pregnancy-associated glycoproteins in milk was also used in pregnancy diagnosis, but they are detected after 28 days of pregnancy [14,15].

MicroRNAs (miRNAs) are non-coding single-stranded RNA only consisting of 21–25 nucleotide sequences that regulate gene expression [16]. The precursor miRNA is a pre-miRNA with a stem–loop structure that is produced from the pri-miRNA transcribed with RNA polymerase II and disconnected via Drosha and RNase III [17]. The pre-miRNA is transported from the nucleus to the cytoplasm and becomes a double-stranded miRNA disconnected via Dicer and RNase III [18]. Double-stranded miRNAs bind to Argonaute 2 (Ago2) protein and become mature single-stranded miRNAs. Mature single-stranded miRNAs combine with target mRNA to either inhibit translation or become disconnected [19]. The miRNAs are thought to regulate certain physiological phenomena by regulating gene expression. Some miRNAs produced within cells are secreted into the extracellular space and circulated in the bloodstream. The miRNAs are stable in blood vessels that have abundant nucleases in the presence of extracellular vesicles (EVs), especially exosomes, high-density lipoprotein-enveloped miRNA, or Ago2 protein, which is involved in the biosynthesis of miRNA [20,21,22]. Exosomes are 30–120 nm nanosized vesicles that play an important role in intercellular communication by carrying proteins, lipids, mRNA, and miRNAs [23]. Exosomes exist in various bodily fluids, such as urine and blood, and certain diseases and treatments can affect their formation. These features render exosomes as potential biomarkers for liquid biopsies. The miRNAs carried by exosomes act as cancer biomarkers [24].

In a previous study, we demonstrated the profile of circulating miRNAs in Japanese Black cows 21 days after AI using microarray analysis [25]. That study was conducted to identify miRNAs that are biomarkers for bovine pregnancy diagnosis, and the miRNAs that differed between pregnant and non-pregnant groups included miR-19b [26], miR-25 [27,28], miR-26b [26,29,30], miR-27b [30], miR-29a [28], and miR-148a [30], which have been previously reported. Previously reported miRNAs [25] may serve as biomarkers for the early diagnosis of pregnancy in cattle. However, the accuracy of the diagnosis could not be ascertained because of the limited number of samples examined.

In the present study, we investigated the levels of prospective miRNA biomarkers to identify early pregnancy markers in cows. Furthermore, we investigated the features of EVs that are thought to transport miRNAs in the blood and whether the miRNAs we focused on were contained in EVs.

## 2. Materials and Methods

### 2.1. Animals

The Iwate University Laboratory Animal Care and Use Committee approved the experimental and feeding conditions for the cattle used in this study (approval numbers A201701 and A202006). Japanese Black (JB) cattle obtained from an experimental farm were used in this study.

### 2.2. Experimental Design

#### 2.2.1. Study 1

A total of 37 samples of approximately 5 mL of peripheral blood were collected into an EDTA tube from cows 21 d after AI. The cows were assigned into the pregnant (P, n = 22) and non-pregnant (NP, n = 15) groups according to the results of pregnancy diagnosis using ultrasonic detection (UD) at 35 d of gestation. Pregnant cows were further divided according to their reproductive history into heifers (n = 5), primiparous (n = 4), and multiparous (2 or more, n = 13) groups. We selected the prospective miRNAs listed in our previous report, including miR-19b, miR-25, miR-27b, miR-29a, miR-30d, miR-126-3p, and miR-148a [25]. Plasma miRNA levels were detected using real-time quantitative reverse transcription polymerase chain reaction (RT-qPCR) and compared between the pregnant and non-pregnant groups. Based on these results, we evaluated the feasibility of early pregnancy diagnosis using miRNAs.

#### 2.2.2. Study 2

**Figure 1:**
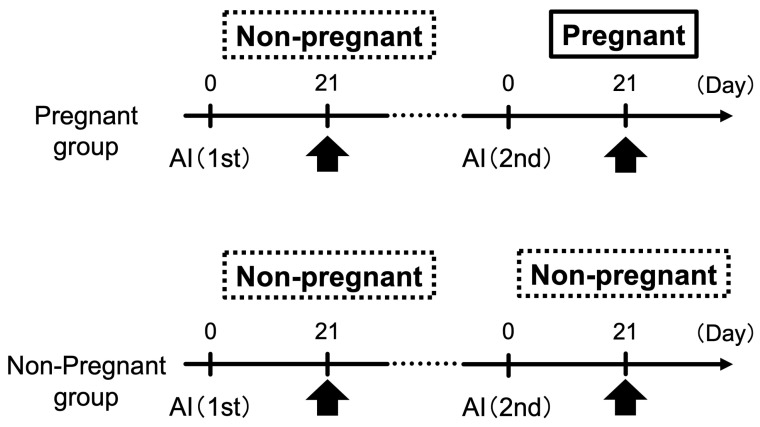
Experimental design of Study 2. Pregnant and non-pregnant cows were sampled on Day 21 of gestation. Arrows indicate blood collection. The seven cows that were not pregnant with the first artificial insemination (AI) (1st) were divided into two groups. The first group included cows pregnant with the second AI (2nd, n = 4) on Day 21, and the second group included those that remained non-pregnant (n = 3).

Study 2 comprised seven cows that were not pregnant during the first AI in Study 1. Blood samples were collected from the cows 21 days after the second AI in both individual cows that became pregnant with the second AI and those that did not (Figure 1). Similar to Study 1, RT-qPCR was performed to investigate the plasma miRNA fluctuations within the same individual cow.

#### 2.2.3. Study 3

Approximately 5 mL of peripheral blood was collected into an EDTA tube. Samples from six individual cows were collected on Day 21 after AI. According to the result of pregnancy diagnosis using UD at 35 d of gestation, cows were categorized into the pregnant (P, n = 3) and non-pregnant (NP, n = 3) groups. The Western blotting of EV marker proteins was performed to investigate the presence of miRNAs in EVs. Once the presence of miRNAs in the EVs was confirmed, the small RNA extraction, RT-PCR, and RT-qPCR of EV miRNA were performed.

### 2.3. miRNA Extraction and RT-qPCR

The miRNAs were extracted from plasma using the miRNeasy Serum/Plasma kit (QIAGEN, Hilden, Germany) according to the manufacturer’s instructions. The extracted miRNA was measured using a Nanodrop-1000 spectrophotometer (Thermo Scientific, Waltham, MA, USA) and reverse-transcribed to complementary DNA (cDNA) using a Mir-X™ miRNA First-Strand Synthesis Kit (TaKaRa, Shiga, Japan), and cDNA was synthesized from 60 ng of miRNA.

RT-qPCR was performed using KOD SYBR qPCR Mix (TOYOBO, Osaka, Japan) and an ABI7300 real-time PCR system (Applied Biosystems, Foster City, CA, USA). The forward primers are listed in Table 1. For the reverse primer, we used the mRQ 3′Primer (TaKaRa, Shiga, Japan), which corresponds to the adapter sequence supplied in the Mir-X™ miRNA First-Strand Synthesis Kit (TaKaRa, Shiga, Japan). The thermal cycling conditions comprised an initial incubation of the sample at 98 °C for 2 min, followed by 40 cycles of 98 °C for 15 s, 60 °C for 10 s, and 68 °C for 30 s. In this study, a standard curve method was used for absolute quantification. To determine the copy number of the miRNAs, a standard curve was generated for each miRNA using a serial dilution of standard oligonucleotides containing the corresponding mature miRNA and adapter sequences. Standard oligonucleotides for RT-qPCR were synthesized by Eurofins Genomics (Tokyo, Japan) and are listed in Table S1. The dissociation curve for the identification of the SYBR Green-based objective amplicon was confirmed, and the quantity of each miRNA was assessed at each peak of the amplicon. Blood miRNA levels were normalized using miR-2455 as the reference gene [25].

### 2.4. Estimation of Threshold Values

A receiver operating characteristic (ROC) curve was constructed based on miRNA expression levels on Day 21 of gestation in AI cows. The Youden index and area under the ROC curve were estimated using JMP 7 software (SAS Institute Inc., Cary, NC, USA). In the context of pregnancy diagnosis in AI cows on Day 21 of gestation, the Youden index was used as the threshold value for prediction accuracy. The classifications of true positives, false positives, true negatives, and false negatives are listed in Table 2. Sensitivity (probability of a pregnancy diagnosis among pregnant cows), specificity (probability that cows were diagnosed as non-pregnant), positive predictive value (probability of a true-positive diagnosis), and negative predictive value (probability of a true-negative diagnosis) were calculated as previously described [13].

### 2.5. Isolation of EVs in Plasma

Using 1 mL of plasma from pregnant cows on Day 21 as the sample, EVs were purified via ultracentrifugation for the nano-tracking analysis (NTA). The purification of EVs and NTA was performed by Theoria Science, Inc. (Chiyoda-ku, Tokyo, Japan) using NanoSight LM10 (Malvern Panalytical, Malvern, UK). To quantify each miRNA in EVs, the EVs were extracted from plasma using a Total Exosome Isolation Kit (from plasma) (Invitrogen, Carlsbad, CA, USA) according to the manufacturer’s instructions. Purified EV pellets were resuspended in the Exosome Resuspension Buffer of the Total Exosome RNA and Protein Isolation Kit (Invitrogen) to obtain an EV solution. The protein concentration of the EV solution was measured using a Quick Start protein assay Kit (Bio-Rad, Hercules, CA, USA) with bovine serum albumin (Nacalai Tesque, Kyoto, Japan) as the standard, according to the manufacturer’s instructions. In addition, small RNA was extracted from EVs using a Toral Exosome RNA and Protein Isolation Kit (Invitrogen) according to the manufacturer’s instructions. The concentration of the extracted small RNA was measured using a Nanodrop-1000 spectrophotometer (Thermo Scientific).

### 2.6. Western Blotting

Western blotting was performed as previously described [31]. The EV proteins were separated using 12% sodium dodecyl sulfate-polyacrylamide gel electrophoresis and transferred to PVDF membranes (Immobilon-P, Millipore Corporation, Bedford, MA, USA). The membranes were blocked with 10% skim milk for 16 h at 4 °C and incubated with a custom-made anti-CD63 antibody (1:1000; Exo AB Antibody Kit, System Biosciences, Palo Alto, CA, USA) for 1 h at room temperature. Membranes were subsequently incubated with goat anti-rabbit HRP secondary antibody (1:20,000; Exo AB Antibody Kit) for 1 h at room temperature. ChemiDoc (Bio-Rad) was used to detect immunoreactive CD63 using the Chemi-Lumi One Luminous Reagent (Nacalai). The intensity was determined via densitometric analysis using the ImageJ 1.53 software “https://imagej.net/ij/ (accessed on 3 November 2020)”. The CD63 protein levels in the EVs of pregnant cows were defined as 1, and protein levels in non-pregnant cows were calculated relative to these.

### 2.7. RT-PCR and Agarose Gel Electrophoresis of EV miRNA

For the RNA obtained from EVs, cDNA was synthesized using the Mir-X™ miRNA First-Strand Synthesis Kit (TaKaRa), and RT-PCR was performed with TaKaRa PCR Thermal Cycler Dice (TaKaRa) using TaKaRa Ex Taq (TaKaRa). The primers were each miRNA-specific primer used in the RT-qPCR and the mRQ 3′Primer supplied with the Mir-X™ miRNA First-Strand Synthesis Kit (TaKaRa). After electrophoresis on a 2% agarose gel, the bands were soaked in ethidium bromide and detected using a Gel Doc EZ (Bio-Rad).

### 2.8. Statistical Analysis

Student’s *t*-test or Wilcoxon’s test was used for comparisons between two different groups, and Student’s *t*-test was used for comparisons between two paired groups. For multigroup comparisons, the Tukey–Kramer test was used. Statistical analyses were performed using the JMP 7 software. Results were classified as statistically significant if their *p*-value was less than 0.05, and trending if their *p*-value was between 0.05 and 0.1.

## 3. Results

### 3.1. Study 1: Circulating miRNA in AI Cows and ROC Curve Analysis

**Figure 2:**
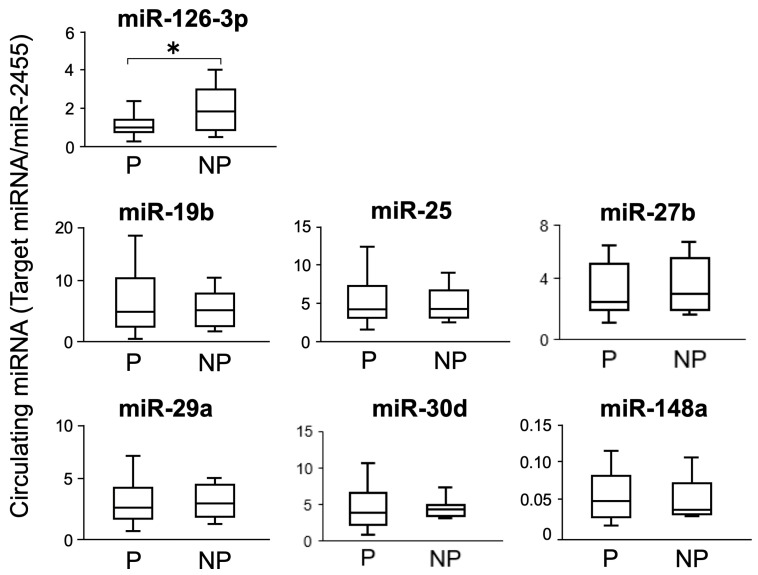
Circulating miRNA levels in pregnant and non-pregnant cows on Day 21 after AI. Plasma miRNA levels of pregnant (P, n = 22) and non-pregnant (NP, n = 15) cows on Day 21 after AI were analyzed using RT-qPCR and normalized to miR-2455 levels. Data are shown as the means ± SEM. Statistical analysis was performed using Student’s *t*-test. Asterisks indicate significant differences between the pregnant and non-pregnant groups (*p* < 0.05).

On Day 21 after AI, the plasma levels of miR-19b, miR-25, miR-27b, miR-29a, miR-30d, miR-126-3p, and miR-148a were analyzed in both pregnant and non-pregnant cows. The expression of miR-126-3p was significantly lower in pregnant cows than in non-pregnant cows; however, the expression of the other six miRNAs did not differ between the two groups (Figure 2).

**Figure 3:**
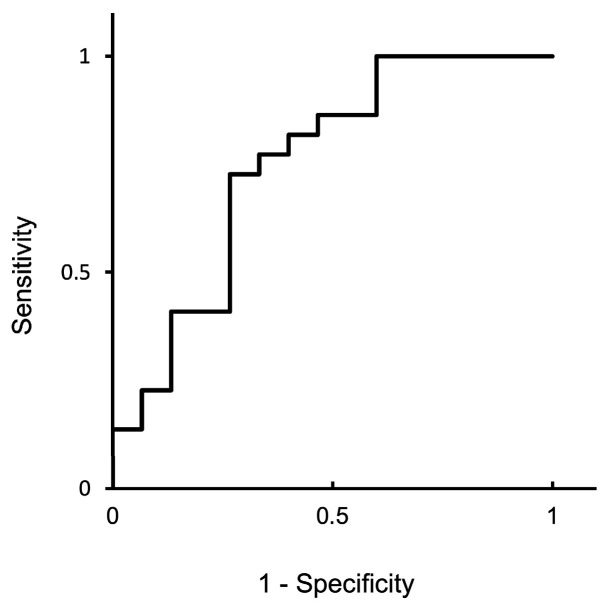
Receiver operating characteristic (ROC) curve of circulating miRNA levels and predictive values in AI cows on Day 21 of gestation. Circulating miR-126-3p levels were analyzed using the ROC curve.

An assessment using an ROC curve was used to statistically confirm whether miR-126-3p is suitable for the detection of early gestation (Figure 3). The calculated cutoff value was 1.25. The area under the curve, which indicated the diagnostic performance of the test, was 0.75, indicating moderate validity. The sensitivity, specificity, positive predictive value, and negative predictive value were 72.7%, 73.3%, 80.0%, and 64.7%, respectively.

**Figure 4:**
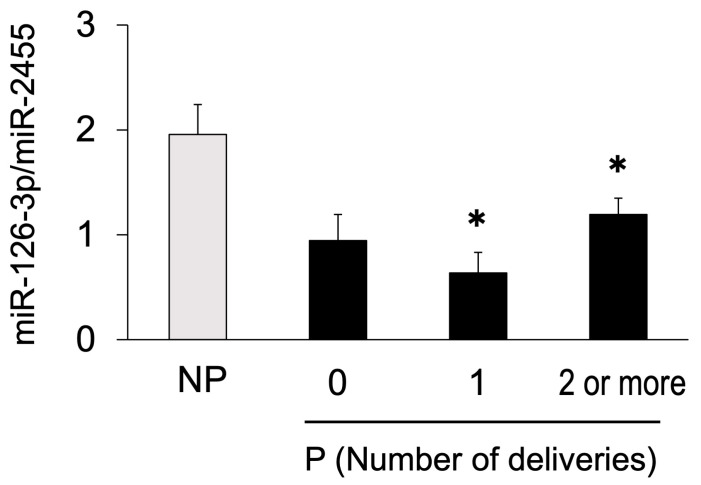
Circulating miR-126-3p levels in pregnant cows for each reproductive history and in non-pregnant cows on Day 21 after AI. Plasma miR-126-3p levels of pregnant (P, n = 22) and non-pregnant (NP, n = 15) cows on Day 21 after AI were analyzed using RT-qPCR and normalized to miR-2455 levels. Pregnant cows were divided according to their reproductive history into heifers (0, n = 5), primiparous cows (1, n = 4), and multiparous cows (2 or more, n = 13). Data are shown as the means ± SEM. Statistical analysis was performed using the Tukey–Kramer test. Asterisks indicate significant differences between the non-pregnant and heifer, primiparous, or multiparous pregnant groups (*p* < 0.05).

To determine whether parity was an effect, we analyzed miR-126-3p levels according to parity: heifers, primiparous, and multiparous. The miR-126-3p levels in primiparous and multiparous cows were significantly lower than those in non-pregnant cows; however, levels in heifers did not differ from those in non-pregnant cows (Figure 4). In contrast, the levels of miR-19b, miR-25, miR-27b, miR-29a, miR-30d, and miR-148a were similar in non-pregnant cows and heifers, primiparous, and multiparous pregnant cows (Figure S1).

### 3.2. Study 2: Changes in Circulating miRNA Levels during Pregnant and Non-Pregnant States in the Same Individual on Day 21 of Gestation

**Figure 5:**
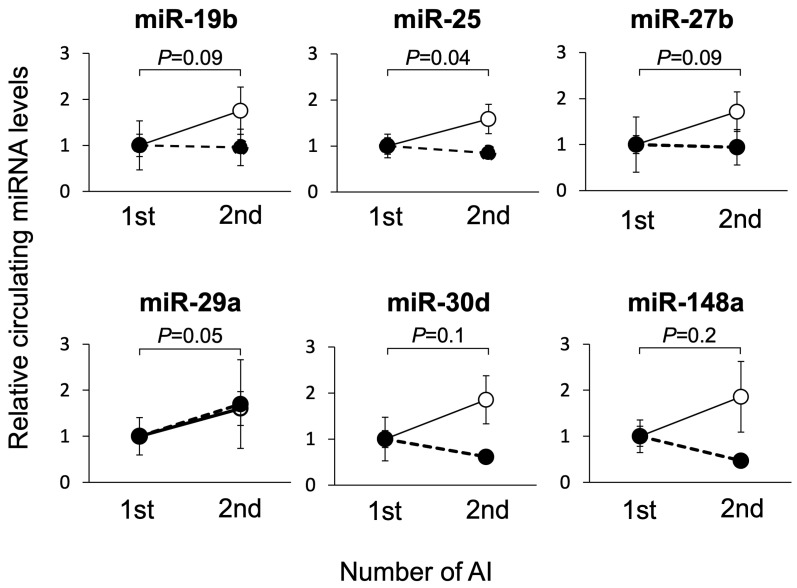
Comparison of circulating miRNA levels in the same individual between pregnant and non-pregnant states on Day 21 of gestation. The cows were divided into a pregnant group (n = 4, open circles) and a non-pregnant group (n = 3, closed circles), as shown in Figure 1. Circulating miRNA levels were analyzed using RT-qPCR. The miRNA level at the first AI (1st) was defined as 1 and relative miRNA levels were calculated for the second AI (2nd) in the same individual cow. Data are shown as the means ± SEM. Statistical analysis was performed using Student’s *t*-test. *p*-values indicate differences for the same individual cow between the non-pregnant state in the first AI group and the pregnant state in the second AI group. No changes in the levels of miRNAs were found between the first and second AI in the non-pregnant group.

To account for the differences in plasma miRNA levels between individual cows at the basal level, we compared the plasma miRNA levels in the same individual cow in the pregnant and non-pregnant groups for the miRNAs except miR-126-3p, which showed no differences between these groups at first AI, as shown in Figure 1 (Figure 5). Among the seven individual cows that were not pregnant at the first AI, the miRNA levels of the group that became pregnant at the second AI (P, n = 4) and those that remained non-pregnant (NP, n = 3) were compared. Blood samples were collected 21 d after AI. In the pregnant group, the miR-25 levels were significantly higher in the second AI group than in the first AI group. The levels of miR-19b, miR-27b, and miR-29a were higher in the second AI group. In the non-pregnant group, there were no changes in the miRNA levels between the first and second AI in the same individual.

### 3.3. Study 3: Detection of Target miRNAs in EVs Isolated from Bovine Plasma

**Figure 6:**
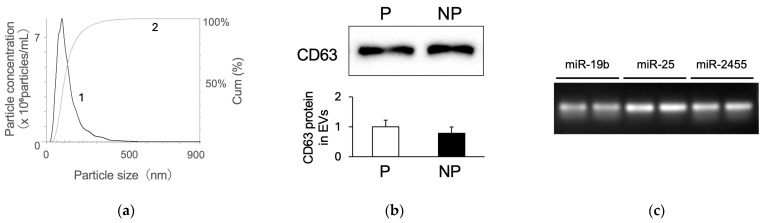
Nanoparticle tracking analysis, detection of EV markers, and target miRNAs in EVs isolated from bovine plasma. (**a**) Particle size distribution in bovine plasma was measured using nanoparticle-tracking analysis. Curve 1 describes the relationship between particle number distribution (left Y axis) and particle size (X axis); curve 2 describes the correlation between cumulative percentage distribution of particles (percentile in right Y axis) and particle size (X axis). (**b**) Detection of CD63, an EV exosome marker protein, in EVs isolated from the bovine plasma of pregnant (P) and non-pregnant (NP) cows was performed using Western blotting. The upper panel indicates the Western blotting image and the lower panel indicates the results of densitometric analysis. The CD63 protein level in EVs of pregnant cows was defined as 1, and the relative protein levels were calculated in non-pregnant cows. Data are shown as the means ± SEM. Statistical analysis was performed using Student’s *t*-test. No changes in the levels of CD63 protein in EVs were observed between the pregnant and non-pregnant groups. (**c**) RT-PCR was used to detect miRNAs in EVs isolated from bovine plasma samples from two pregnant cows.

To clarify the mode of existence of miRNAs in bovine plasma, we detected EVs in bovine plasma and miRNAs in these EVs. The plasma of pregnant cows was analyzed using NTA, and particles approximately 100–150 nm in size were observed, which is equal to the diameter of EVs (Figure 6a). The detected particle sizes were 131 nm on average, with most particles being 95 nm in size. Western blotting analysis showed that CD63, a marker protein for EV, was present in the EV fraction from the plasma of both pregnant and non-pregnant cows (Figure 6b). The expression levels of CD63 did not differ between the two groups (Figure 6b).

**Figure 7:**
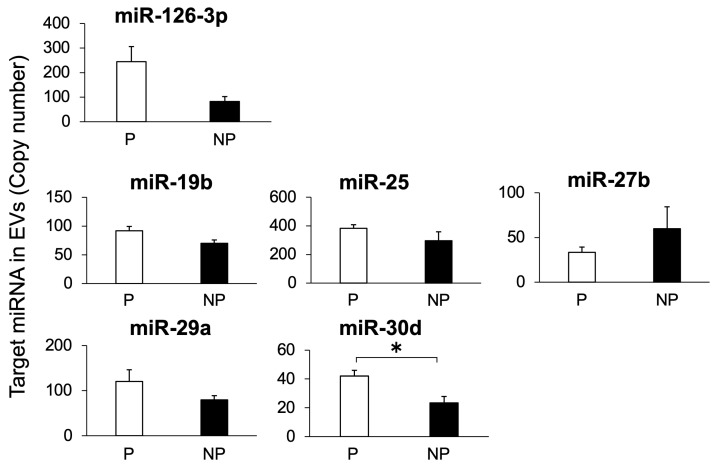
Target miRNA levels in EVs isolated from plasma of pregnant and non-pregnant cows on Day 21 after AI. The miRNA levels in EVs isolated from the plasma of pregnant (P, n = 3) and non-pregnant (NP, n = 3) cows 21 days after AI were analyzed using RT-qPCR. Data are shown as the means ± SEM. Statistical analysis was performed using Student’s *t*-test. Asterisks indicate significant differences between the pregnant and non-pregnant groups (*p* < 0.05).

RT-PCR was performed on three miRNAs, miR-19b, miR-25, and miR-2455, in descending order of plasma content to evaluate their presence in EVs; all three were found to be present (Figure 6c). RT-qPCR was performed for all seven circulating miRNAs (Figure 2) to determine their levels in EVs (Figure 7). The presence of miR-148a was not detected in the EV fraction. The six other miRNAs were detected in the EVs, and miR-30d levels were higher in pregnant cows than in non-pregnant cows. The presence of miR-2455 was also detected in EV, and as with plasma, no differences were observed between pregnant and non-pregnant cows (Table S2). For the six miRNAs detected in EVs, we compared their levels in plasma and the EVs for the same individual; the levels were lower in plasma than in EVs for all six miRNAs (Table S2).

## 4. Discussion

Circulating miRNAs change owing to physiological phenomena or diseases [32]. As biomarkers for the diagnosis of diseases, miRNAs must be chosen based on the condition of a specific change owing to the target disease. In this study, based on a previous report [25], we determined the circulating miRNA levels on Day 21 after AI to identify additional potential biomarkers for early pregnancy in cows.

Six of the seven miRNAs did not differ significantly between the pregnant and non-pregnant cows. However, the expression of miR-126-3p was found to be significantly lower in pregnant cows than in non-pregnant cows, which may prove to be a valuable diagnostic tool for gestation. In contrast, we previously reported no differences in miR-126-3p levels between pregnant and non-pregnant cows [25]. We cannot explain the reason for this; however, the number of samples may have been limited in the previous study. 

We evaluated the accuracy of the diagnosis of gestation based on circulating miR-126-3p levels. The area under the curve calculated via ROC analysis was 0.75, and the accuracy was moderate. Therefore, it was important to identify non-pregnant cows in the subsequent AI. The diagnosis of early gestation requires high specificity; however, the specificity of miR-126-3p was only 73.3% in the present study. In addition, the feasibility of this diagnostic method must be assessed using additional samples to determine its viability for practical use.

Circulating miRNAs in cows change owing to the effect of genetic factors and age [33]. Therefore, we divided the pregnant cows according to reproductive history into heifers, primiparous cows, and multiparous cows, and compared the miRNA levels between each group of pregnant and non-pregnant cows to consider the effect of parity. In the primiparous and multiparous pregnant groups, miR-126-3p was significantly lower than in the non-pregnant group, whereas the other six miRNAs were not affected by parity. Thus, miR-126-3p may only be used as a biomarker of parous cows.

Detected in various animal species, miR-126-3p is involved in immunity and angiogenesis [34]. Additionally, miR-126-3p suppresses metastatic cancer formation by inhibiting the proliferation of human breast cancer [35]. No relevance to pregnancy has been reported, and additional examination is needed regarding the mechanism underlying the decline in the plasma miR-126-3p levels of pregnant cows.

The cows shown in Figure 2 included non-pregnant individuals at the first and second AI. We examined circulating miRNA levels in the same individual cow at different time points (Figure 5). In the four cows that were not pregnant at the first AI and became pregnant at the second AI, miR-25 was significantly higher at the time of pregnancy than in non-pregnant cows, and miR-19b, miR-27b, and miR-29a showed similar trends. In contrast, among the three cows that were not pregnant at the first AI and remained non-pregnant at the second AI, miRNA levels, except for that of miR-29a, did not show an increasing trend. These results support the findings of previous studies on miR-19b [26], miR-25 [27,28], and miR-27b [30]. However, we could not confirm a significant change when we compared pregnant and non-pregnant cows without considering individual variability. It is possible to use miRNAs for the diagnosis of gestation by considering the comparison method, for example, to establish a baseline for individual cows.

Circulating miRNA envelopes in exosomes correspond to EVs [20]; therefore, we attempted to detect EVs in the plasma of cows and the miRNAs in those EVs. The average diameter of the particles isolated from the bovine plasma was 131 nm. This is consistent with the previously reported features of exosomes [36]. Western blot analysis detected CD63, a marker protein for EVs, in the EV fraction from the plasma of both pregnant and non-pregnant cows. The CD63 levels did not differ between the two groups. According to these results, the level of EVs in the plasma did not differ between pregnant and non-pregnant cows. Additionally, the seven miRNAs were identified, and reference miRNAs were also detected in EVs in plasma, revealing that these miRNAs exist in EVs.

When the levels of the various miRNAs in plasma exosomes were compared with those of miRNAs in whole plasma, the levels of all miRNA molecular species in the exosomes were extremely low compared with those in whole plasma, with some being below the detection limit. Although most miRNAs circulating in plasma are reported to be contained in exosomes, which are EVs [20], in the present study, the amount of miRNA detected in plasma exosomes was much smaller than that detected in whole plasma. In contrast, the main carriers of miRNAs circulating in the plasma are lipoproteins such as high-density lipoprotein and Ago2 proteins, in addition to EVs such as exosomes, which depend on miRNA [37]. Therefore, these results suggest that most of the miRNAs measured in this study may be carried by carriers other than exosomes, and to confirm this, miRNAs must be extracted from high-density lipoprotein and Ago2 proteins purified from plasma, the levels measured in the same manner, and compared. Furthermore, exosome purification is one reason for the low miRNA levels extracted from exosomes. The most common methods for exosome purification are ultracentrifugation, which simply precipitates pellets containing exosomes, and density gradient centrifugation, in which a gradient is formed depending on the particle type [38]. In addition, immunoprecipitation using antibodies against exosome surface proteins, such as CD63; ultrafiltration using particle size separation; and precipitation using reagents containing polymers used in the present study are recommended methods [23]. Although the yield of exosomes obtained by ultracentrifugation is high, the machine is difficult to operate, and particles other than exosomes are often mixed. Although the precipitation method using polymer reagents is simple, it has the disadvantage that the yield of exosomes obtained is low [39]. Therefore, the recovery rate of exosomes in plasma was low in this study, and the levels of various miRNAs extracted from the exosomes may have been reduced. In addition, the fact that the kit protocol used in this study purified small RNA, including miRNAs, may be a reason for the reduced levels of various miRNAs obtained from exosomes. In bovine plasma, miRNAs account for approximately 70% of the small RNA [29]. These results suggest that the extraction of total miRNAs from plasma is necessary when performing a diagnosis using miRNAs from blood.

## 5. Conclusions

The levels of miR-126-3p were lower in pregnant cows than in non-pregnant cows on Day 21 after AI, and plasma miRNA could be a candidate biomarker for pregnancy diagnosis. However, in the present study, the accuracy of pregnancy diagnosis based on circulating miR-126-3p levels was moderate. In addition, the expression of some miRNAs tended to be higher during the pregnancy state than the non-pregnancy state in the same individual, suggesting that they could be used as an index to determine pregnancy and non-pregnancy rates using a comparative method. As the level of miRNA in EVs was low and difficult to measure, total miRNA should be measured in plasma for diagnosis.

## Figures and Tables

**Table 1:** List of primers used for RT-qPCR.

miRNA	Accession No.	Sequence (5′-3′)
bta-miR-19b	MIMAT0004337	ACACGTTTAGGTTCGTTTTGACTAAA
bta-miR-25	MIMAT0003853	GTAACGTGAACAGAGCCAGACTAAA
bta-miR-27b	MIMAT0003546	AAGTGTCACCGATTCAAGACGAAA
bta-miR-29a	MIMAT0003518	GATCGTGGTAGACTTTAGCCAATAAA
bta-miR-30d	MIMAT0003533	ACATTTGTAGGGGCTGACCTTCGAAAA
bta-miR-126-3p	MIMAT0004328	GTAATAATGAAAACCATGCGCAAA
bta-miR-148a	MIMAT0003522	AGTCACGTGATGTCTTGAAACAAAA
bta-miR-2455	MIMAT0012037	AGACACGAGCCCCTCCGTCCCTAAA

**Table 2:** Classification of true positives, false positives, true negatives, and false negatives.

Status	miR-126-3p Values *^1^	Ultrasonic Detection *^2^
True positives, TP	+	+
False positives, FP	+	−
True negatives, TN	−	−
False negatives, FN	−	+

Pregnancy status was determined as positive or negative using the cutoff value of the Youden index for miR-126-3p on Day 21 after AI and ultrasonic detection at approximately 60 days of gestation. *^1^ In the miR-126-3p values column,”+” and “−” indicate values higher and lower than the cutoff value, respectively. *^2^ In the ultrasonic detection column, “+” and “−” indicate a positive and negative pregnancy diagnosis by ultrasonography 60 days after AI, respectively.

## Data Availability

The original contributions presented in this study are included in the article/Supplementary Materials. Further inquiries can be directed to the corresponding author.

## Supplementary Materials

The following supporting information can be downloaded at: https://www.mdpi.com/article/10.3390/ani14111592/s1, Table S1: List of standard oligonucleotide sequences synthesized for RT-qPCR; Table S2: Plasma miRNA and exosome miRNA levels of pregnant and non-pregnant cows on Day 21 after AI; Figure S1: Circulating miRNA levels in pregnant cows based on reproductive history and in non-pregnant cows on Day 21 after AI.
