# Supplementary material for: Potential of Circulating miRNA Biomarkers and Exosomes for Early Pregnancy Diagnoses in Cattle
Source: Animals (Basel). 2024 May 28;14(11):1592. doi: 10.3390/ani14111592 (PMC11171103; doi:10.3390/ani14111592)
Supplement: Supplementary file 1 [file animals-14-01592-s001.zip › animals-3024636-supplementary.pdf]

animals

Article

Potential of Circulating miRNA Biomarkers and Exosomes for Early Pregnancy Diagnosis in Cattle

Chiaki Ninomiya[1], Hitomi Yoshino[2], Toshina Ishiguro-Oonuma[1,2], Kosuke Iga[3], Tomomi Kanazawa[1], Toru Takahashi[1,2] and Keiichiro Kizaki[1,2,*]

Table S1. List of standard oligonucleotide sequences synthesized by RT-qPCR.

miRNA	Sequence (5′-3′)	Bases
bta-miR-19b	AGGCAGTGGTATCAACGCAGAG-<br>TACTTTTTTTTTTTTTTTTTTTTTTTTTTTTTTTCAG-<br>TTTTGCATGGATTTGCAGA	78
bta-miR-25	AGGCAGTGGTATCAACGCAGAG-<br>TACTTTTTTTTTTTTTTTTTTTTTTTTTTTTTTTCAGAC-<br>CGAGACAAGTGCAATG	77
bta-miR-27b	AGGCAGTGGTATCAACGCAGAG-<br>TACTTTTTTTTTTTTTTTTTTTTTTTTTTTTTT-<br>GCAGAACTTAGCCACTGTGAA	76
bta-miR-29a	AGGCAGTGGTATCAACGCAGAG-<br>TACTTTTTTTTTTTTTTTTTTTTTTTTTTTTTTTAAC-<br>CGATTTCAGATGGTGCTAG	78
bta-miR-30d	AGGCAGTGGTATCAACGCAGAG-<br>TACTTTTTTTTTTTTTTTTTTTTTTTTTTTTTAGCTTCCAGTCGGG<br>GATGTTTACA	79
bta-miR-126-3p	AGGCAGTGGTATCAACGCAGAG-<br>TACTTTTTTTTTTTTTTTTTTTTTTTTTTTTTCGCGTAC-<br>CAAAAGTAATAATG	76
bta-miR-148a	AGGCAGTGGTATCAACGCAGAG-<br>TACTTTTTTTTTTTTTTTTTTTTTTTTTTTTTACAAAGTTCTGTAG<br>TGCACTGA	77
bta-miR-2455	AGGCAGTGGTATCAACGCAGAG-<br>TACTTTTTTTTTTTTTTTTTTTTTTTTTTTTTTCCCTGCCTCCCCG<br>AGCACAGA	77

Table S2 Plasma miRNA and exosome miRNA levels of pregnant and non-pregnant cows on Day 21 after AI.

miRNA	NP/P	Whole plasma miRNA (copy number)	Exosomal miRNA (copy number)	Exosomal miRNA (%)
miR-19b	NP	27,258 ± 343	92 ± 13	0.34
	P	27,697 ± 8355	70 ± 10	0.25
miR-25	NP	37,141 ± 4336	383 ± 44	1.03
	P	43,446 ± 14,506	296 ± 108	0.68
miR-27b	NP	14,415 ± 812	33 ± 10	0.23
	P	17,687 ± 5,744	60 ± 42	0.34
miR-29a	NP	20,178 ± 4,,609	120 ± 45	0.60
	P	27,909 ± 8852	79 ± 16	0.28
miR-30d	NP	34,558 ± 10,965	42 ± 7	0.12
	P	39,017 ± 18,397	23 ± 8	0.06
miR-126-3p	NP	12,023 ± 4,174	245 ± 107	2.03
	P	9,043 ± 3,089	83 ± 35	0.91
miR-2455	NP	10,850 ± 1369	258 ± 45	2.38
	P	18,873 ± 19243	324 ± 154	1.72

The miRNA levels in the plasma of pregnant (P, n = 3) and non-pregnant (NP, n = 3) cows on Day 21 after AI were analyzed using RT-qPCR. The levels indicate the copy numbers of 1 ng of each miRNA or small RNA. Data are shown as mean ± SD. The percentage of exosomal miRNAs relative to total plasma miRNAs is also indicated.

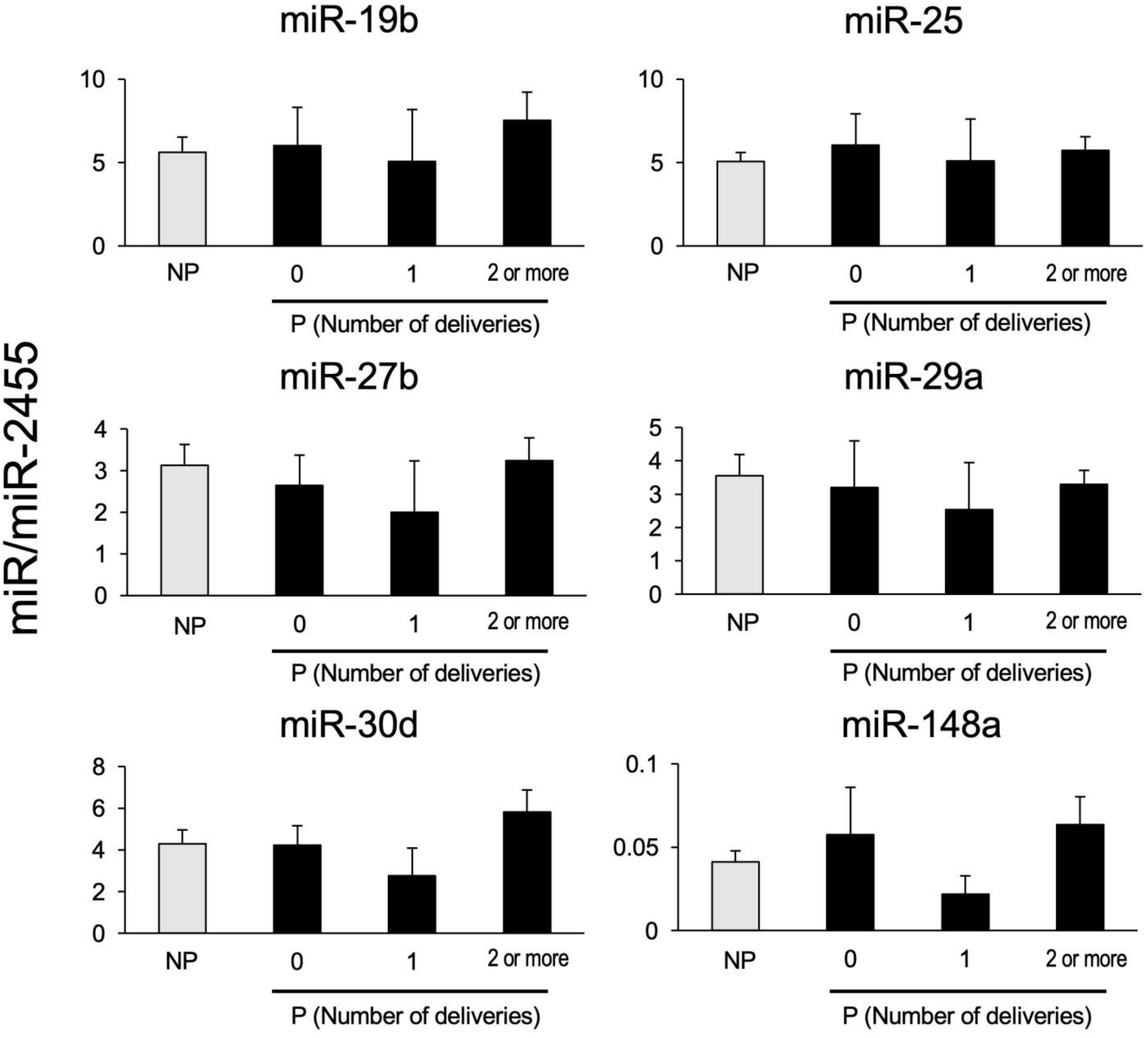

Figure S1. Circulating miRNA levels in pregnant cows for each reproductive history and in non-pregnant cows on Day 21 after AI. The miRNA levels in the plasma of pregnant (P, n = 22) and non-pregnant (NP, n = 15) cows on Day 21 after AI were analyzed using RT-qPCR and normalized to miR-2455 levels. Pregnant cows were divided according to their reproductive history into heifers (0, n = 5), primiparous (1, n = 4), and multiparous (2 or more, n = 13) cows. Data are shown as the mean ± SEM.
